# Descemet membrane endothelial keratoplasty (DMEK): clinical results of precut versus surgeon-cut grafts

**DOI:** 10.1007/s00417-020-04901-7

**Published:** 2020-08-26

**Authors:** Doreen Koechel, Nicola Hofmann, Jan D. Unterlauft, Peter Wiedemann, Christian Girbardt

**Affiliations:** 1 Dept. of Ophthalmology, University Hospital Leipzig, Liebigstraße 10-14, 04103 Leipzig, Germany; 2 German Society for Tissue Transplantation, Feodor-Lynen-Straße 21, 30625 Hannover, Germany

**Keywords:** Descemet membrane endothelial keratoplasty, Precut tissue, Corneal transplantation, Eye bank

## Abstract

**Purpose:**

This study aims to investigate possible differences in clinical outcomes between precut and surgeon-cut grafts for Descemet membrane endothelial keratoplasty (DMEK).

**Methods:**

142 consecutive patients who underwent DMEK were included in the study. 44 patients received precut tissues, and 98 patients received surgeon-cut tissues. Precut grafts were allocated to the patient by the German Society for Tissue Transplantation if available. We compared the outcomes of both groups for changes in visual acuity, central corneal thickness, endothelial cell density, re-bubbling rate, and graft failure rate.

**Results:**

Patients who received precut tissues experienced similar increase in visual acuity (median change 0.4 logMAR) and decrease of corneal swelling (median change 132 μm) compared with those who received surgeon-cut tissues (median VA change 0.3 logMAR, *p* = 0.55, CCT change 118 μm, *p* = 0.63). There was no statistical difference in endothelial cell density (1436 vs. 1569 cells/mm^2^, *p* = 0.37), re-bubbling (32% vs. 35%, *p* = 0.85), and graft failure rate (5% vs. 1%, *p* = 0.23). No primary graft failure occurred in the group of precut grafts.

**Conclusion:**

Both methods lead to comparable results for visual acuity, corneal deswelling, endothelial cell density, and re-bubbling rate. A previously described higher graft failure rate for precut tissues could not be confirmed in our study. Thus, we do not see medical reasons against the use of precut tissues. There are several advantages of precut DMEK tissues over surgeon-cut tissues, especially the prevention of graft loss during preparation in the operating theater.

## Introduction


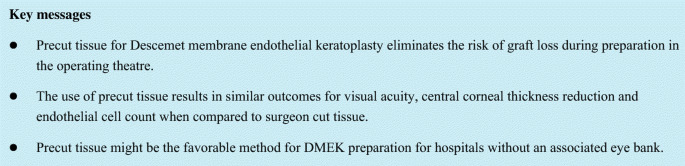
Since its first description by Melles [1] in 2006, the overwhelming success of Descemet membrane endothelial keratoplasty (DMEK) has helped many patients with corneal endothelial disorders to obtain better vision and increased quality of life. DMEK consists of stripping off Descemet’s membrane with the adherent endothelium from a donor eye and transferring it as a roll into the anterior chamber of the patient’s eye subsequent to the removal of that eye’s Descemet’s membrane. The graft is then unfolded and fixed by an air or gas bubble.

Besides graft unfolding, the most challenging step is graft preparation directly before the operation. Therefore, the idea of preparing the graft as much as possible in the tissue bank was developed [2]. The aim of these precut grafts is standardized preparation under aseptic conditions by experienced staff and therefore prevention of graft loss during preparation in the operating theater. The latter is especially relevant because of widespread graft shortage. Further advantages of precut tissues are shorter duration for transplantation, delivery on schedule ready for use, and microbiological surveillance after preparation [3].

Those advantages are especially relevant in eye hospitals without an associated eye bank, i.e., centers that do not have the possibility of switching to another graft in case of preparation failure and tissue loss.

In Germany, precut tissues have been provided by the German Society for Tissue Transplantation, Hannover, since December 2015.

In this study, we searched for possible differences in clinical outcomes of patients who received a precut tissue compared with patients whose graft was prepared by the surgeon directly before DMEK in the operating theater.

## Material and methods

### Patients

 142 consecutive patients who received DMEK at the Leipzig University Eye Hospital between April 2016 and December 2017 were included. All grafts were supplied by the German Society for Tissue Transplantation seated in Hannover, Germany. The study was performed as an observational cohort study. Whenever possible, precut tissues were used. Due to limited availability of suited corneas for pre-preparation, not every request for precut tissues could be fulfilled. 44 patients received precut tissues. The other 98 patients received surgeon-cut tissues. All grafts were allocated by the German Society for Tissue Transplantation as available, without knowledge of individual patient parameters. Thus, a pseudo-randomization was achieved.

Operation was performed as DMEK alone (*n* = 74) for pseudophakic patients or in combination with phacoemulsification and posterior bag lens implantation (triple DMEK, *n* = 68) for all phakic patients.

The study adhered to the tenets of the Declaration of Helsinki, and the approval for the study was obtained from the Ethics Committee of the Medical Faculty of Leipzig University.

### Surgeon-cut tissues

Donor’s eyes were retrieved by the German Society for Tissue Transplantation in accordance with the German transplantation law after obtaining tissue donation consent and evaluation of the donor medical history within 72 h after cardiovascular arrest. The eyes were transported to the tissue bank cooled in a sterile moist container, where they were trephined with a diameter of 16 mm as a corneoscleral disc. Subsequent organ culture was performed in cornea culture medium K1 (Merck Millipore®, Darmstadt, Germany) at a temperature between 31 and 37 °C. Before transplantation, donor corneas were deswollen in culture medium K2 (Merck Millipore®, Darmstadt, Germany), consisting of medium K1 with addition of 6% dextran 500. The culture period was median 22 days with a minimum of 11 days for surgeon-cut and 14 days for precut corneas and maximum 34 days for both types of corneas including 1 to 6 days deswelling time. All tissue manipulations were performed under sterile conditions in laminar air flow with no-touch technique. Microbiological testing of both culture medium and donor tissue was performed, and negative results were mandatory before further processing of the grafts. After verification of sufficient endothelial cell quantity and morphology, grafts were sent to Leipzig University Eye Hospital. In the operating theater, preparation of the Descemet’s membrane via liquid bubble technique as described by Szurman [4] was performed: Iris base was identified, where a sharp dissection up to Schwalbe’s line was conducted. Further preparation of a tunnel was made with a blunt spatula until approximately 2 mm central of Schwalbe’s line. A liquid bubble with trypan blue was injected directly into the sub-Descemet space. Circular trephination was performed. If liquid bubble technique could not be performed successfully, as an alternative, the technique described by Cursiefen and Kruse [5] was performed: Descemet’s membrane was circularly scratched and then nearly completely lifted toward the center with two forceps. Trephination was performed, stripping of Descemet’s membrane was completed, and the graft was stained with trypan blue. After preparation, the graft was transferred into a glass injector.

### Precut tissues

The procedures performed at the German Society for Tissue Transplantation until delivery to Leipzig were identical to those described above. Additionally, trephination was performed, and Descemet’s membrane with endothelium was nearly completely dissected from the stroma, leaving only a central area adherent [6]. Such prepared tissues were sent to Leipzig University Eye Hospital in dextran-containing medium K2. In the operating theater, tissue was washed with balanced salt solution, and the adherent central portion of the graft was completely dissected from the underlying stroma. The graft was transferred into a glass injector.

### DMEK

After preparation, standard DMEK was performed in both groups as initially described by Melles [1]. All surgeries were performed by C.G. and J.D.U. When combined with phacoemulsification and posterior bag lens implantation, those steps were performed before the actual DMEK. At the end of the operation, 20% sulfur hexafluoride gas was injected into the anterior chamber. All operations took place under general anesthesia.

### Postoperative management

Patients were advised to lie flat on their backs postoperatively. If significant graft detachment was detected via OCT within the first postoperative days, re-bubbling with 20% sulfur hexafluoride gas was performed. If necessary, this was repeated until complete graft attachment was achieved. Medical treatment consisted of 3 days of intravenous prednisolone as well as topical steroids, antibiotics, and pilocarpine. Topical steroids were tapered monthly and kept once per day for 1 year after operation.

Follow-up visits took place 1, 3, 6, and 12 months after operation. Further visits were performed if necessary, especially if immunological reaction or graft failure was suspected. Primary graft failure was defined as permanently persistent corneal edema immediately after DMEK despite correct graft orientation. Secondary graft failure was defined as corneal edema with subsequent deterioration of vision after an initial period of a clear cornea, without observation of any signs of graft rejection or improvement after topical steroids.

Postoperative data for visual acuity and central corneal thickness (CCT) were taken from the follow-up visit 6 months after operation. CCT was measured by corneal tomography (Pentacam® HR, Oculus®, Wetzlar, Germany). Endothelial cell density (ECD) measurements were not routinely performed in all patients. If available, ECD data were taken from the follow-up visit 6 months after operation. ECD was measured using specular microscopy (CEM-530, Nidek® Co. Ltd., Tokyo, Japan).

### Statistical analysis

Visual acuity, CCT as measured via OCT, ECD, re-bubbling rate, and graft failure rate in both groups were analyzed. In a subgroup analysis, possible differences for these parameters between DMEK and triple DMEK cases were analyzed. Best-corrected visual acuity was converted from decimal to logMAR. Statistical analysis was performed with SPSS® Statistics Software Version 21.0 (IBM®_,_ Armonk, NY). Categorical variables are presented as numbers and compared with Fisher’s exact test, while continuous variables are presented as median (1st–3rd quartiles) and compared with two-sided*t* test. A significant result was defined as *p* < 0.05. Diagrams were created with Excel® 2013 (Microsoft®, Redmond, WA).

## Results

### Patient data and donor characteristics

Patient and donor characteristics for both groups are given in Table 1. No differences were seen in both groups preoperatively.

**Table 1:** Patient characteristics for precut and surgeon-cut tissue recipients

	Precut	Surgeon-cut	*p*
Number of eyes (*n*)	44	98	
Age (years)	72.0 ± 8.1	71.5 ± 8.3	0.79
Gender (f/m)	33/11	60/38	0.11
Phakic/pseudophakic	18/26	56/42	0.11
Status post-capsulotomy	5	13	0.29
Visual acuity (logMAR)	0.5 (0.4/0.7)	0.4 (0.3/0.7)	0.39
CCT (μm)	651 (588/681)	627 (597/675)	0.9
Donor ECD (cells/mm^2^)	2497 ± 212	2465 ± 232	0.51
Donor age (years)	69.0 ± 8.5	73.0 ± 9.8	0.08
Donor gender (f/m)	16/28	40/58	0.25

### Visual acuity

**Fig. 1:**
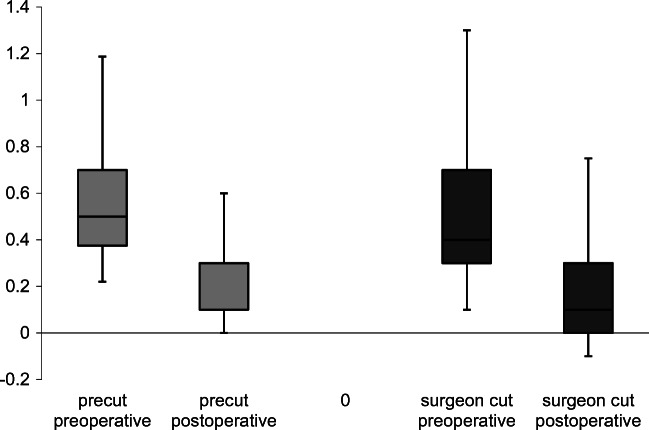
Increase of visual acuity (decrease of logMAR) after 6 months

We found no significant difference in development of visual acuity between patients who received precut tissues and those with surgeon-cut tissues. Median change in visual acuity is 0.4 logMAR in the precut group and 0.3 logMAR in the surgeon-cut group (*t* test, *p* = 0.55; Fig. 1, Table 2).

**Table 2:** Visual acuity, central corneal thickness and endothelial cell count median and quartiles (Q1/Q3), re-bubbling, and graft failure rates before and 6 months after operation

	Precut	Surgeon-cut	*p*
Visual acuity (logMAR)	0.1 (0.1/0.3)	0.1 (0/0.3)	0.55
CCT (μm)	519 (497/559)	509 (488/546)	0.63
ECD (cells/mm^2^)	1436 (1161/1857)	1569 (1323/1907)	0.37
Re-bubbling	14 (32%)	34 (35%)	0.85
Graft failure	2 (5%)	1 (1%)	0.23

### Central corneal thickness

**Fig. 2:**
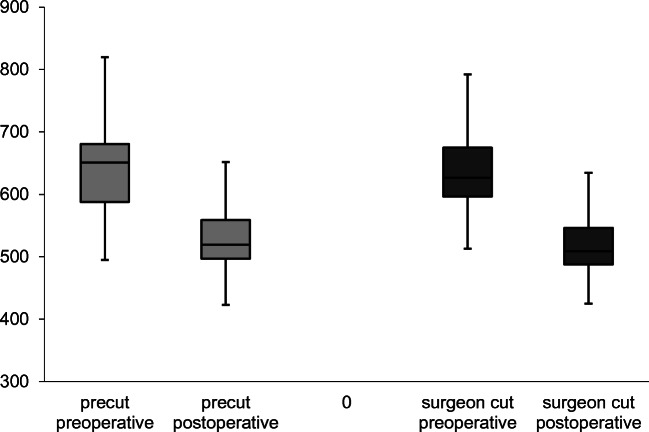
Decrease of central corneal thickness after 6 months

The changes in CCT before and after transplantation did not show a significant difference between the two groups. Median change in CCT is 132 μm in the precut group and 118 μm in the surgeon-cut group (*t* test, *p* = 0.63; Fig. 2, Table 2).

### Endothelial cell density

Data from ECD measurements 6 months after operation were available from 59 patients, with 20 in the precut and 39 in the surgeon-cut group. Median ECD was 1436 cells/mm^2^ in the precut group and 1569 cells/mm^2^ in the surgeon-cut group. There is no significant difference between the two groups (*t* test, *p* = 0.37; Table 2).

### Re-bubbling

We determined whether re-bubbling procedures after transplantation were needed. We find no significant difference between both groups (Fisher’s exact test, *p* = 0.85; Table 2).

### Graft failure

We experience graft failures in both groups (Table 2). There are two graft failures in the precut group and one in the surgeon-cut group (Fisher’s exact test, *p* = 0.23). Both graft failures in the precut group were secondary graft failures. The first one appeared 16 months after operation and the second one 21 months after operation. Successful re-DMEK was performed in both cases. The graft failure in the surgeon-cut group was a primary graft failure, i.e., despite correctly orientated and attached graft swelling of the patient’s cornea persisted. The case could be rescued with successful re-DMEK as well.

### Complications

No significant difference was found in complications between the two groups. Postoperative intraocular pressure elevation was seen in 4 cases (9%) in the precut group and in 6 cases (6%) in the surgeon-cut group (Fisher’s exact test, *p* = 0.5). Postoperative macular edema was seen in 3 cases (7%) in the precut group and in 2 cases (2%) in the surgeon-cut group (Fisher’s exact test, *p* = 0.17). There was one (2%) immunologic graft reaction in the precut group and no one in the surgeon-cut group (Fisher’s exact test, *p* = 0.3).

### DMEK versus triple DMEK

In a subgroup analysis, we compared changes in visual acuity, CCT, ECD, re-bubbling rate, and graft failure between patients who received DMEK alone and patients who received triple DMEK. Similarly to the results shown above, there are no significant differences for change in visual acuity, ECD, and re-bubbling rate (Table 3).

**Table 3:** Subgroup analysis of results for DMEK versus triple DMEK procedures

	Precut DMEK/triple DMEK	Surgeon-cut DMEK/triple DMEK	*p*
Visual acuity increase (logMAR)	0.3/0.4	0.4/0.4	0.85/0.74
CCT reduction (μm)	140/117	202/109	0.16/0.73
ECD (cells/mm^2^)	1609/1429	1399/1704	0.47/0.19
Re-bubbling	8/6	15/19	0.79/1.0
Graft failure	2/0	1/0	0.55/1.0

CCT reduction showed a significant difference between DMEK and triple DMEK (*p* < 0.05) with a greater reduction in the DMEK group. This might be due to the fact that this group comprised pseudophakic bullous keratopathy cases with primarily decompensated corneas. No differences in CCT reduction were found between precut and surgeon-cut tissues within the groups (Fisher’s exact test, *p* = 0.16 and 0.73, respectively).

The above-mentioned three graft failures all occurred in the DMEK group. No graft failure occurred in the triple DMEK group. However, Fisher’s exact test showed no significant difference (*p* = 0.55).

## Discussion

Time disjuncture of graft preparation and the actual DMEK procedure have some advantages over graft preparation directly before the operation. Eye hospitals with associated eye bank have the possibility of pre-stripping donor tissue and keeping it in organ culture, which does not seem to affect clinical outcomes [7]. However, eye hospitals without an associated eye bank are dependent on preparation and delivery of corneal grafts by external eye banks. Even when performed by experienced staff, DMEK preparation has a small but constant failure rate, probably due to an abnormal Descemet’s membrane-stromal interface [8]. Precut tissues offer the possibility of higher standardization in preparation and avoid the risk of graft loss directly before the operation.

The current study compared clinical outcomes of patients who received precut grafts and patients who received surgeon-cut grafts. Our results suggest that the well-known benefits of DMEK as described in larger cohorts [9–11] can be achieved by in-site preparation as well as with precut grafts. This was established for a much larger sample size than previously investigated.

Our results contradict the previously published results on clinical outcomes of precut DMEK tissues: An observational retrospective cohort study from Freiburg, Germany, found a higher number of graft failures in their precut group. It has to be mentioned that the group which received precut tissues consisted of only 11 patients, while the surgeon-cut group consisted of 453 patients. Precut tissues were prepared at the same site, but preparation was stopped after trephination, and the graft was stored in a deswelling organ culture medium for 1–2 days. The authors suspected the second storage in deswelling media with high molecular dextran and the short storage in saline solution as reason for their results [12].

A retrospective comparative study from Lyon, France, investigated 11 patients that received precut tissues and 22 patients that received surgeon-cut tissues. There was no significant difference in CCT and ECD between both groups 6 months after surgery. There was one graft failure with precut tissues and four graft failures with surgeon-cut tissues [13].

Prospective data are available from a study of 22 patients that received precut tissues and 29 patients that received surgeon-cut tissues. Precut tissues were prepared in the eye bank 1 day before transplantation and stored in organ culture media. Surgeon-cut tissues were prepared directly before surgery. The statistical analysis of both groups showed comparable results for visual acuity outcomes, change in CCT, endothelial cell loss, and graft failure rate [14]. Comparable with that study, our data showed no significant differences in clinically relevant outcomes as well. It is worth mentioning that both cases of graft failure in our precut group were secondary graft failures (after 16 and 21 months, respectively). It is difficult to imagine any effect in differences of graft preparation after such a long time. In fact, our graft failure rate matches the known graft failure rate as described by other groups [15, 16]. We therefore do not see any relation with graft preparation mode.

There are some limitations of our study: Allocation to either of both study groups was not performed via true randomization. However, precut grafts were provided by the German Society for Tissue Transplantation whenever possible without knowledge of individual patient parameters. Specular microscopy was not performed routinely in all patients, so ECD data were not available for all patients.

Presently, widespread use of precut graft is limited mainly because of two practical reasons: Limited availability due to higher work load in the eye bank and significant higher costs. With further acceptance, both factors might be overcome, and the advantages of precut tissues might set the path for them as standard in DMEK preparation for hospitals without an associated eye bank.
